# Facilitators and challenges in recruiting pregnant women to an infant obesity prevention programme delivered via telephone calls or text messages

**DOI:** 10.1186/s13063-018-2871-5

**Published:** 2018-09-15

**Authors:** Mahalakshmi Ekambareshwar, Seema Mihrshahi, Li Ming Wen, Sarah Taki, Greer Bennett, Louise A. Baur, Chris Rissel

**Affiliations:** 1 Sydney School of Public Health, Sydney Medical School, University of Sydney, Sydney, Australia; 2 NHMRC Centre of Research Excellence in the Early Prevention of Obesity in Childhood, Sydney School of Public Health, University of Sydney, Sydney, Australia; 3 Charles Perkins Centre, University of Sydney, Sydney, Australia; 4 Health Promotion Unit, Sydney Local Health District, Sydney, Australia; 5 Discipline of Child & Adolescent Health, University of Sydney, Sydney, Australia; 6 New South Wales Office of Preventive Health, Ministry of Health, Sydney, Australia

**Keywords:** Recruitment, Trials, Pregnant women, Process evaluation, Facilitators, Challenges, Telephone, Text messages

## Abstract

**Background:**

Recruitment of pregnant women into trials is a challenge exacerbated by a number of factors, including strict eligibility criteria. There has been little in-depth examination of the recruitment process to trials involving pregnant women. This paper presents the findings of a study conducted to identify facilitators and challenges in recruiting pregnant women to the Communicating Healthy Beginnings Advice by Telephone (CHAT) randomised controlled trial, which aims to reduce the prevalence of infant and childhood obesity.

**Methods:**

Data were collected from (1) administration of a short questionnaire to women at the time of recruitment exploring women’s reasons for consent and non-consent; (2) interviews with recruiters to capture recruiters’ experiences of the recruitment process; and (3) analysis of field notes taken by recruiters on the number of women approached/recruited and reasons as to why they did not consent to participate. Data obtained were triangulated to gain insights into the process of recruiting pregnant women.

**Results:**

A total of 1155 pregnant women (mean gestational age 31.5 weeks) were enrolled over 5 months. The main reasons for women consenting to participate in the study were convenience in programme delivery mode via telephone calls or text messages, altruism and because the programme was free of charge. The main reasons for women not consenting were lack of interest, language challenges/difficulty speaking English and some felt they did not need information and support due to prior experience as a mother. Facilitators included organisational support, rapport with recruiters and some women with no other children who needed advice. Despite the challenges, the mode of delivery of intervention via telephone calls or text messages, the minimal effort required of women to participate, organisational support from the lead site and recruiters’ knowledge of and commitment towards the trial contributed towards successful recruitment.

**Conclusion:**

Despite some challenges in recruiting pregnant women to an infant obesity prevention programme, some of the facilitators in recruitment included mode of delivery of the intervention programme via telephone calls or text messages, the minimal effort required for women to participate, organisational support from the lead site, and recruiters’ knowledge of and commitment towards the trial.

**Trial registration:**

The CHAT RCT is registered with the Australian Clinical Trial Registry (ACTRN12616001470482p); Ethics Review Committee of Sydney Local Health District (Protocol No. X16–0360 & LNR/16/RPAH/495).

## Background

Recruitment of participants to trials is acknowledged as a challenging facet of research [1–4]. The success of most participatory research and trials primarily depends on effective recruitment and retention of trial participants [5]. Trials that experience difficulties in recruiting participants have had to extend their recruitment period, vary their recruitment strategy or, in some cases, modify their eligibility criteria in order to achieve adequate sample sizes [6–9].

In the case of pregnant women, recruitment is more challenging to researchers due to the narrow window of eligibility within which to recruit and deliver the intended intervention [10, 11], time constraints on women with competing priorities [5, 12, 13], work commitments [10, 14], responsibilities of caring for other children [9], and disinterest in research [9, 15].

To date, a comprehensive process evaluation of the recruitment of pregnant women to a low intensity health promotion trial for infant obesity prevention has not been undertaken. This paper focused on the trial recruitment process of the Communicating Healthy Beginnings Advice by Telephone Randomised Controlled Trial (CHAT RCT) [16], to identify facilitators and challenges in recruiting pregnant women to a low intensity health promotion programme where health behaviour change messages are delivered via telephone calls or text messages. Recruitment was explored from the perspectives of pregnant women (consenting and non-consenting) and recruiters. The detailed documentation of the recruitment process will assist with translation and replication or for scaling-up of infant obesity prevention trials and interventions to a population level.

## Methods

### Study setting

The CHAT RCT was conducted across four Local Health Districts within New South Wales in Australia, namely Sydney, South Eastern, South Western and Southern New South Wales Local Health Districts. Pregnant women were recruited at eight hospital sites within the above districts between February 23 and July 27, 2017. The main method of recruitment was opportunistic recruitment at the antenatal clinic waiting rooms by appointed recruiters (three full-time, two part-time and one casual). The CHAT RCT study made use of the opportunity to recruit pregnant women at the time women came to their routine antenatal clinic appointments at the eight hospital antenatal clinics.

### Eligibility criteria

Women were eligible to participate in the CHAT RCT if they were 18 years old and over, were in the third trimester of their pregnancy (28–34 weeks gestational age), were able to communicate in English, had a mobile phone and lived in the recruitment area. Women were ineligible if they had a severe medical condition, could not give informed consent, were expecting multiple births or their babies were expected to have major foetal anomalies.

### Ethical considerations

The CHAT RCT is registered with the Australian Clinical Trial Registry (ACTRN12616001470482p) on October 21, 2016; Ethics Review Committee of Sydney Local Health District (Protocol No. X16–0360 & LNR/16/RPAH/495).

### Study design

Data were collected and analysed from the following three sources: (1) administration of a short questionnaire to women at the time of recruitment to explore women’s reasons for consent and non-consent; (2) interviews with recruiters to capture recruiters’ experiences of the recruitment process; and (3) analysis of field notes taken by the recruiters on the number of women approached and recruited, and the reasons as to why women did not consent to participate.

#### Phase 1 – Short questionnaire at time of consent

The first data collection phase involved administration of short multiple response and open-ended questions at the time of recruitment to women who consented and to women who did not. Data capture for this phase commenced on June 13, 2017, until the conclusion of trial recruitment on July 27, 2017. The brief questionnaire was administered at the end of the consenting process to consenting women, while non-consenting women were asked if they would please answer the brief questions.

Pregnant women who consented to participate completed three optional questions on Research Electronic Data Capture (REDCap) [17] using recruiters’ hand-held tablets. REDCap is an electronic data capture tool and a secure web-based application on which the CHAT RCT consent and participants’ data were obtained and captured. Women had the option to choose multiple reasons from a list provided or to provide ‘other’ reasons for participation.

Pregnant women who did not consent to participate were also requested to complete a brief optional questionnaire. Women had the option to choose multiple reasons from a list provided or to provide ‘other’ reasons for non-participation. Data captured on REDCap were exported to Microsoft Office Excel for analysis.

#### Phase 2 – Interviews with recruiters

Interviews were conducted face-to-face with four recruiters (by ME) during the recruitment phase of the trial to explore their experience of the recruitment process. Recruiters provided written informed consent. Interviews were audio-recorded using a digital recorder and downloaded as voice files, and were between 18 and 30 min in duration. Interviews were semi-structured to explore emerging themes [18], using an interview guide with seven questions.

To reduce researcher bias, voice files were listened to, coded and analysed independently by two researchers (ME and SM) using the broad research questions as an initial coding framework. Themes were then discussed by the researchers for comparison and consistency of coding, with dominant themes identified and mutually agreed upon. Direct interview quotations illustrate key themes but recruiters’ names were de-identified as recruiters A to D.

#### Phase 3 – Analysis of field notes

Recruiters wrote hand-written field notes on a purpose-built spreadsheet at point of recruitment. The field notes contained information on recruitment date, recruitment location, number of women who were approached, number of women who consented, number of women who did not consent and recruiters’ perspective of reasons for women not consenting to the trial. The handwritten field notes were later transferred to a combined single Excel spreadsheet. These data were transferred to a Structured Query Language database for analysis of numbers of women approached, numbers of women who did not consent, and recruiters’ perspective of women’s reasons for not consenting. Recruiters’ field notes on women’s reasons for not consenting were grouped into ten broad categories, of which the top four categories were ‘not interested’, ‘not first baby’, ‘limited or no English’ and ‘need more time/information’.

Data collected from the three recruitment phases were triangulated for validation, a strategy that has the benefit of raising the research above narrow interpretations and personal biases that stem from single methodologies [19].

## Results

### Recruitment and enrolment into CHAT RCT

**Fig. 1:**
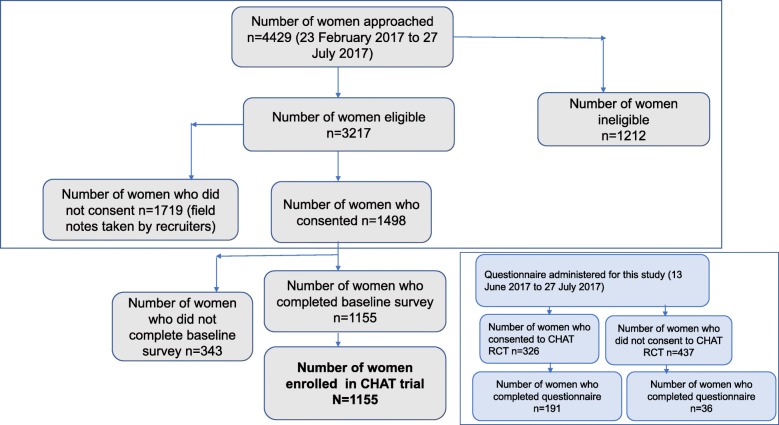
CHAT RCT recruitment flow diagram and number of women (consenting and non-consenting) who completed the questionnaire

A total of 1498 out of 3217 eligible women (47%) who were approached consented to participate in the CHAT RCT. Recruitment at antenatal clinics while women waited for their scheduled appointments proved to be a successful recruitment strategy, with 1155 women successfully recruited to the CHAT RCT within the stipulated timeframe, which was more than the anticipated number of 1056 women (Fig. 1).

### Characteristics of participants

Of those who completed the questionnaire for consenting women, 67% were above 30 years of age; 63% of women lived in a household with income ≥ AUS$ 80,000; almost half (48%) were having their first child (primiparous). Characteristics of participants who completed the questionnaire for consenting women were similar to those of the overall CHAT RCT participants, wherein 68% were > 30 years of age, 55% had a household income ≥ AUS$ 80,000 and 54% were primiparous.

### Reasons for participation provided by consenting women at time of recruitment

Although recruitment to the CHAT RCT was undertaken over 5 months, the short purpose-built questionnaire was administered to participants between June 13 and July 27, 2017. During this period, 326 women consented to participate in the CHAT RCT. Of the 326 women, 191 (59%) completed the questionnaire administered to consenting women. Reasons for participation are provided in Table 1; multiple responses were allowed.

**Table 1:** Reasons for participation provided by 191 consenting women at time of recruitment

Reasons for participation in study provided by consenting women	Number of women who chose this reason, *n* (%) Number of responses (*n* = 239)
Information by SMS	79 (33)
Information by telephone	71 (30)
Altruism	41 (17)
Free programme	17 (7)
Information seeking	17 (7)
Seeking help/guidance	8 (3)
Incentives offered	6 (3)

Overall, 63% of responses indicated that women consented due to the convenient mode of programme delivery via text messages (33%) and telephone calls (30%), 17% of responses indicated that women consented due to altruism to help with research or to help other women, and 7% indicated that they participated since it was free of charge (Table 1). Women’s responses and interest in receiving information via telephone calls or text messages were an indication that the trial might have value for them.

### Reasons for non-participation provided by non-consenting women at time of recruitment

Between June 13 and July 27, 2017, 437 women did not consent to participate in the CHAT RCT. Of the 437 women, only 36 (8%) women completed the short purpose-built questionnaire administered to non-consenting women since non-consenting women were already not interested. Hence, triangulation of data was undertaken and data gathered from recruiters’ field notes on women’s reasons for not consenting at point of recruitment was analysed. Of the few women who completed the questionnaire, one-fifth indicated language barriers and difficulty speaking English, and a further one-fifth indicated that they were not primiparous. Other reasons for non-participation were being busy and not needing information and support (Table 2). Other characteristics of women (such as age, income and parity) are not known since data were not collected from these women.

**Table 2:** Reasons for non-participation provided by 36 non-consenting women at time of recruitment

Reasons for non-participation provided by non-consenting women	Number of women who chose this reason, *n* (%) Number of responses (*n* = 47)
Not first baby	10 (21)
Limited or no English	10 (21)
Busy	8 (17)
Do not need information or support	7 (15)
Not interested	6 (13)
Need more time/information	3 (6)
Unwilling to participate in research/survey	3 (6)

### Reasons for non-participation from recruiters’ field notes

Recruiters documented the reasons for non-participation for 1719 women. These were grouped and consolidated into eleven categories, of which the top three reasons for non-participation were women not being interested, women not being primiparous, and women speaking limited or no English (Table 3). Of those women who did not consent due to language barriers (238) and for whom language was known (194), the most common languages were Mandarin, spoken by 71 women (37%), followed by Cantonese spoken by 28 women (15%).

**Table 3:** Reasons for non-participation from recruiters’ field notes at time of recruitment

Reasons for non-participation	Number of women, *n* (%)
Not interested	804 (47)
Not first baby	386 (23)
Limited or no English	238 (14)
Need more time/information	122 (7)
Moving away from recruitment area	37 (2)
Do not need information or support	31 (1.8)
Recruitment incomplete – women called	31 (1.8)
Too busy	29 (1.7)
Not well/tired	18 (1)
Unwilling to participate in research/survey	13 (< 1)
Other reasons	10 (< 1)

### Interviews with recruiters

Quotes from qualitative interviews conducted with recruiters are tabulated (Table 4). There was consensus that recruitment declined over time due to (1) the narrow window of eligibility within which to recruit pregnant women; (2) new women not meeting the eligibility criteria (although new women came to the clinics, these women were very early in their pregnancy with gestational age less than the CHAT RCT’s eligibility criteria of 28–34 weeks’ gestational age); and (3) reaching saturation within the clinics from where women were recruited (e.g. the same women coming in for their routine monthly, fortnightly or weekly antenatal clinics as these women had already been approached). Women’s interest declined due to women seeing recruiters more often as frequency of women’s visits to the antenatal clinics increased with gestational age. The process of approaching women became easier with time for recruiters; however, repeating the same information led to recruitment fatigue (quote 1; Table 4).

**Table 4:** Quotes on recruiters’ perception of recruitment from interviews with recruiters

Quote #	Recruiters’ comments
1	“*It has gone quite tedious recruiting, because you are saying the same things over and over again. Mentally and physically exhausted by the end of the week and recover on weekend*” (Recruiter C)
2	“*The study is so beneficial, incentives involved, not much effort from participant*” (Recruiter B)
3	“*Organisational support that we have in the study, where we have the hospitals to recruit. The managers of the hospitals, they already know that we are going there to recruit, makes our life easier. Going to clinics and having one on one with each participant makes it easier. The organisation is making it easier, it would be impossible to recruit more than a thousand women (as we did) without having the help of so many hospitals and being part of an organisation*” (Recruiter A)
4	“*Rapport with women is the most important. I was able to gain rapport with women by explaining the study, being very clear and transparent, not hiding anything about the study helped me gain women’s rapport*” (Recruiter B)
5	“*I can really talk about childbearing and childrearing and I can practically say that I did not have this information when I had my child and I know that as a first-time mum if I had this information it would have really helped me and that convinces a lot of people. Being a mum does have a positive influence*” (Recruiter C)
6	“*At two recruitment sites you have a high number of people from non-English speaking background, they readily related to me and especially when I started speaking their language with the women, I gained their trust*” (Recruiter B)
7	“*Some women feel like they do not have the time to commit to the study and to complete the survey, they do not need the extra support*” (Recruiter B)
8	“*I have had to convince women who have had children already, I did some convincing and said the information might serve as a reminder*” (Recruiter B)
9	“*After explaining the programme to women, some women still do not really understand it, so you sit down next to them and explain a little longer*” (Recruiter D)
10	“*The word ‘study’ scared some people, using the word ‘programme’ and explaining to them that the ‘programme’ has been going for quite a long time, we are just trying to make it more cost effective, they then trusted it. They did not want to feel like guinea pigs in a ‘study’. Using the word ‘programme’ is how we overcame a barrier*” (Recruiter D)
11	“*I recruited across all eight sites. At two non-metropolitan locations women are not so open to participating because they do not understand the significance of research and how it could benefit them. General lack of understanding of research. Women with higher education levels were more open to research. The two rural hospitals were not aware of significance of research and benefits*” (Recruiter B)
12	“*None of the women declined to participate in the study due to randomisation*” (Recruiter B)
13	“*My interest in the study got them interested, it showed on my face. I was able to gain rapport with women by explaining the study, being very clear and transparent, not hiding anything about the study helped me gain women’s rapport*” (Recruiter B)
14	“*Script was a guide, but did not use exact words since I considered it unnatural. I benefited more from watching other recruiters initially*” (Recruiters C and D)

The following themes were identified from the interview transcriptions: facilitators, challenges, research awareness and variation between sites and recruiters, concept of randomisation, and prior research experience and training.

#### Facilitators to recruitment

The mode of intervention delivery via telephone calls or text messages was convenient to women and hence conducive to recruiting pregnant women, women did not need to travel and minimal effort was required of participants, which made participation easier (quote 2; Table 4). Organisational support provided by the existing antenatal clinic staff was also seen as a facilitator (quote 3; Table 4).

Recruiters considered building rapport with women and gaining the trust of women as highly important. Other facilitators included similar cultural backgrounds and characteristics of the recruiters. For example, one recruiter commented that being older and also being a mother assisted women (quote 5; Table 4). Women from non-English speaking backgrounds related to recruiters who spoke their native language, which helped build trust (quote 6; Table 4), noting that an ability to communicate in English was still necessary to take part in the study.

Recruiters perceived that the study attracted more women who were primiparous, although both primiparous and multiparous women who had family members (husband or mother) with them were encouraged by the family members to participate. Women without family support and women who were new to the country were also attracted to this trial and responded well. Some women consented to the study because they wanted to help other mothers.

Other factors that recruiters perceived as facilitators were that information provided was free of charge and that the information provided in the booklets served as a reference for women. Some women identified with the Raising a Healthy Baby poster displayed at antenatal clinics, associating the project with the poster, which increased their interest. Multiparous women who had difficulty with breastfeeding their previous babies considered that the breastfeeding advice in this trial might be beneficial to them. Recruiters believed that prior introduction of the study by nurses or midwives would have raised women’s awareness of this study and attracted greater numbers of women to the study. Additionally, recruiters believed women would have identified with the study if recruiters had worn a T-shirt with the name of the study.

#### Challenges in recruitment

A major challenge during recruitment was the use of the word ‘study’. Women associated negative connotations to the word ‘study’ (quote 10; Table 4). Some women associated the word ‘study’ with being used as “*guinea pigs*” (quote 10; Table 4). Recruiters commented that, when women were informed of surveys as data collection tools, some women believed they would not have time to complete the telephone surveys (quote 7; Table 4). Some women considered the survey as invasive and did not wish to provide income and personal details. Women who had previous children were also hesitant to participate in the trial since they considered that they had enough experience and did not need information or support; these women often needed further explanation of the potential benefits of the study by recruiters to participate (quote 8; Table 4). Lack of awareness of the research process (e.g. randomisation) and potential benefits by some women from low educational and low socioeconomic status made it difficult for recruiters (quote 9; Table 4).

Further challenges to recruitment were language barriers of women from non-English speaking backgrounds, women moving interstate or overseas, being a younger woman and being affected by obesity. In general, most women did not want to have a conversation but just wanted to complete consent forms when approached.

#### Research awareness and variation between sites

Recruiters agreed that there were differences between sites and between recruiters. Socioeconomic status, awareness of research and cultural factors influenced pregnant women’s decision to participate. At recruitment sites with greater numbers of women from high socioeconomic status, women were open to new experiences and had knowledge and understanding of research. At recruitment sites with greater numbers of women from low socioeconomic background and sites where women were comparatively younger, women were less open and did not see any benefits to participating in the study (quote 11; Table 4).

Variable methods of approaching women were used at different sites. For example, at one recruitment site, terms such as ‘raising a healthy baby’ and ‘nurse help’ appealed to women whereas the term ‘childhood obesity’ was stigmatising. In general, women associated negatively with ‘childhood obesity’ in comparison to ‘healthy baby’. Recruiters noted that, at two non-metropolitan recruitment sites, women responded well to the word ‘support’.

#### Randomisation

Most women did not seem concerned by the idea of being randomised into a particular arm of the study (of three possible arms: telephone support, text message support and usual care) (quote 12; Table 4). While receiving calls from nurses appealed to few women, many did not want to receive telephone calls and preferred to receive text messages. All women were pleased to receive the information booklets.

#### Prior research experience and training

The recruiters’ prior recruitment experience and thorough knowledge of the study were helpful in recruiting pregnant women to this RCT. Recruiters considered this particular study’s recruitment experience was easier than others due to the absence of clinical tests and procedures. Recruiters’ knowledge of and interest in the study and their clear explanation of the study to women were perceived as important factors in recruitment (quote 13; Table 4). Recruiters who commenced with the study prior to trial commencement were involved in drafting recruitment materials and protocols. Subsequent recruiters had the opportunity to learn by watching recruiters who were already recruiting. This was seen as an effective way of learning, with recruiters’ perception that watching recruiting styles of more than one recruiter would have enhanced the learning.

## Discussion

We conducted a process evaluation of recruiting pregnant women to a low intensity health promotion trial for an early childhood obesity prevention trial to identify facilitators and challenges that would contribute to the knowledge base on successful recruitment of pregnant women. The purpose of providing a thorough review of the recruitment process is two-fold, first, for the purpose of replication if translated elsewhere and, second, for scaling-up of the trial to a population level.

Recruitment at antenatal clinics while women waited for their scheduled appointments has proved to be a successful recruitment strategy in other studies [1, 11, 20]. In this trial, 1155 women were successfully recruited to the CHAT RCT within the stipulated timeframe, which was more than the anticipated number of 1056 women [16]. The similarity of participants’ characteristics in this study sample to that of the overall CHAT RCT participants suggests that participants’ reasons for consenting to participate are representative of all women who consented to participate in this trial.

Several of the factors that enabled recruitment were similar to those outlined in the literature on recruitment of pregnant women and new mothers to health promotion programmes. Facilitators were the free-of-charge nature of the programme [21], benefit to women who have had no previous children [22], altruism [9, 13, 23], organisational support to conduct the study and to undertake recruitment at antenatal clinics [9–11, 24], and the recruiters’ ability to establish rapport with women [7, 9, 10, 23, 24]. However, facilitators unique to the success of this recruitment process were the convenience in programme delivery mode via telephone calls or text messages and the nature of the interventions that were not too onerous on women. Converging evidence from all three data collection phases identified that pregnant women considered the mode of intervention delivery via telephone calls or text messages as convenient to them for participation in this trial. Further, pregnant women considered that the trial was not too burdensome and hence were willing to participate. A future challenge is in the subsequent engagement and retention of these women.

Many of the challenges were also similar to those identified in the literature, namely a narrow window of eligibility within which to recruit pregnant women [10, 11], women’s unwillingness to participate in research [15, 23], lack of time to complete the survey [5, 12, 13] and women’s concerns about providing personal information due to privacy issues [9, 25]. Challenges pertinent to this trial were that women felt they did not need information or support since they had previous children, the trial did not appeal to women from low socioeconomic backgrounds and language barriers of women from non-English speaking background [9]. Recruiters tried to overcome some of these challenges, but this was not always possible.

Of the 3217 women who were eligible to participate in the CHAT RCT, 1498 women consented to participate in the study, indicating that the CHAT RCT appealed to almost half of women who were eligible. Those women who consented to participate in the CHAT RCT and completed the short purpose-built questionnaire indicated their participation in the study was due to the convenient mode of programme delivery via telephone calls or text messages and suggested that the trial might have value for them.

Only 8% of pregnant women who did not consent to participate in the CHAT RCT completed the questionnaire for non-consenting women and hence their reasons for non-participation might not be representative of all non-consenting women. The low completion rate of the questionnaire (8%) administered to non-consenting pregnant women was similar to that in another study [15]. However, completion rates may not be comparable to those in many other studies because, in our study, the questionnaire was administered at point of recruitment to explore women’s reasons for not consenting whereas in other studies reasons were explored post-recruitment [8, 12, 14]. For ethical reasons, the CHAT RCT did not collect demographic or personal information from non-consenters. In order to offset this low response rate, triangulation of these data with the other two data collection phases was undertaken. The inability of some women to converse in English was a major challenge, which prompted further analysis of language spoken by non-consenting women [9]. Funding constraints precluded employment of sufficient research staff who spoke other languages; implementation strategies should ensure importance of non-English speaking staff, where possible, since language barriers could be more challenging during scaling-up of health promotion interventions into existing services.

### Strengths and limitations

A key strength of this research was obtaining the perception of recruitment from those closely associated with the recruitment process (consenting women, non-consenting women and recruiters) and at the time of recruitment. Further, converging evidence through triangulation of the data obtained adds to the strength of this study.

While the mode of intervention delivery via telephone calls or text messages appealed to the vast majority of women, there may be women who are not technologically capable and therefore there is a potential risk of not including them, though we believe this group to be negligible. There was limited participation by those women who did not consent to participate in the CHAT RCT and chose not to complete the short purpose-built questionnaire. These women’s views are not included. There is also a risk of excluding women who do not have the financial capacity to maintain a mobile telephone plan that enables two-way communication with the trial team. Another limitation was the inability of some women to converse in English, which precluded them from participating in this trial.

To address the potential for bias or partiality on the part of the researcher, we used different techniques and methods, including triangulation to cross check findings [18, 26], and the scrutiny of material by other researchers.

### Recommendations

Recruiters believed that the introduction of the study to women by nurses and midwives prior to trial commencement would have allowed women to become familiar with the study and strengthened recruitment efforts [10, 15, 23]. Cultural translation of booklets to other languages and the delivery of intervention in languages other than English would benefit women from non-English speaking backgrounds and their children.

## Conclusion

Facilitators and challenges in recruiting pregnant women to an infant obesity prevention programme were identified through process evaluation of the recruitment phase of the CHAT RCT. Pregnant women demonstrated enthusiasm in participating in the CHAT trial, a low intensity health promotion trial for infant obesity prevention. They were recruited at antenatal clinics while waiting for their scheduled appointments. Despite some challenges in recruiting pregnant women to the CHAT RCT, some of the facilitators in recruitment included mode of delivery of the intervention programme via telephone calls or text messages, the minimal effort required of women to participate, organisational support from the lead site, and recruiters’ knowledge of and commitment towards the trial. Pregnant women’s interest in receiving information via telephone calls or text messages was an indication that women valued the trial.

## Abbreviations

CHAT: Communicating Healthy Beginnings Advice by Telephone
RCT: Randomised Controlled Trial
REDCap: Research Electronic Data Capture
